# Exploring Topochemical Oxidation Reactions for Reversible Tuning of Thermal Conductivity in Perovskite Fe Oxides

**DOI:** 10.1021/acs.chemmater.4c02023

**Published:** 2024-10-09

**Authors:** Noa Varela-Domínguez, Marcel S. Claro, Enrique Carbó-Argibay, César Magén, Francisco Rivadulla

**Affiliations:** † Centro Singular de Investigación en Química Biolóxica e Materiais Moleculares (CIQUS), Departamento de Química-Física, Universidade de Santiago de Compostela, 15782 Santiago de Compostela, Spain; ‡ International Iberian Nanotechnology Laboratory (INL), Avenida Mestre José Veiga s/n, 4715-330 Braga, Portugal; § Instituto de Nanociencia y Materiales de Aragón (INMA), CSIC-Universidad de Zaragoza, 50009 Zaragoza, Spain

## Abstract

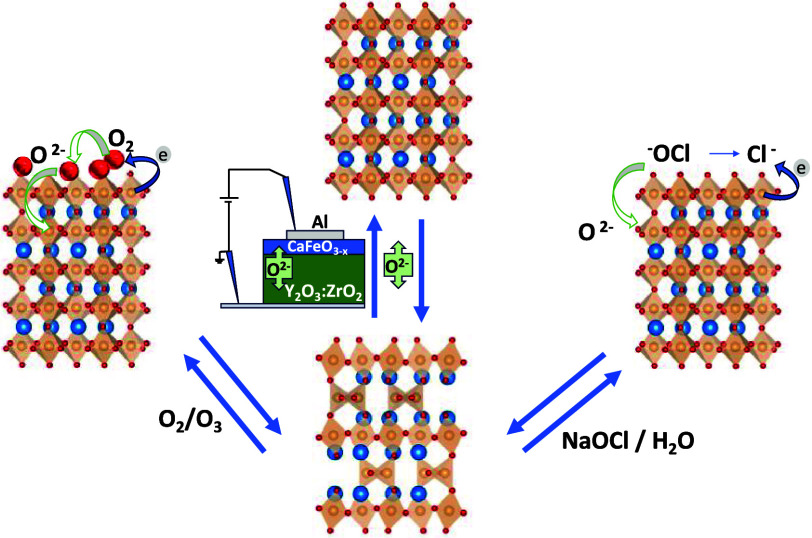

We present a study on the reversibility of thermal conductivity in iron oxides through topochemical oxygen exchange between brownmillerite (BM) (Ca,Sr)FeO_2.5_ and perovskite (PV) (Ca,Sr)FeO_3.0_. By using different oxidation methods, including gas phase (O_2_/O_3_), liquid phase (NaOCl in H_2_O), and solid electrolyte (Y_2_O_3_:ZrO_2_), we demonstrate that the oxidation pathway has a critical influence on the reversibility of the ionic-exchange process. Cyclic oxidation and reduction using O_2_/O_3_ or NaOCl lead to an important accumulation of structural defects, undermining the reversibility of thermal conductivity. In the case of wet oxidation, we demonstrate an inherent tendency of negative charge-transfer oxides toward amorphization and elucidate the origin of this effect. Conversely, the electrochemical injection of the O^2–^ ions via a Y_2_O_3_:ZrO_2_ solid electrolyte reduces structural damage significantly, enhancing both reversibility and durability. This study underscores the importance of selecting appropriate topochemical oxygen exchange methods to maintain structural integrity and optimize functional performance in oxide-based tunable devices.

## Introduction

The flexibility of the oxidation state of transition-metal ions allows the selective O^2–^-anion intercalation/deintercalation in an existing crystalline oxide lattice while maintaining the cationic structure invariant. These are the so-called topotactic or topochemical transformations,^1^ which have been extensively used to modify the magnetic, electric, catalytic, and optical properties of materials in a reversible way.^2−9^

Recently, this strategy has been used to tune the thermal conductivity, κ, of La_1–*x*_Sr_*x*_CoO_3-δ_, through ionic gel gating.^10,11^ Although a large change in κ was achieved by this method, some of the films underwent severe corrosion and dissolution, which resulted in partial irreversibility and reproducibility issues.^10^ In a different approach, Yang et al.^12^ used a solid electrolyte Y_2_O_3_:ZrO_2_ (YSZ) at 280 °C for reversible oxygen insertion of O^2–^ into SrCoO_3−δ_, obtaining a much better cyclability.

In any case, the progressive loss of crystalline order and the accumulation of defects during the repeated topotactic transformations are common to different oxides.^13−17^ Therefore, although topochemical O^2–^-ion exchange is presented as a mild chemical process, normally assumed to be fully reversible, this is not true in general; the strength of the oxidant and the kinetics of the process may influence very much the resulting stability of the structure, compromising the cyclability and therefore the viability of oxide-based tunable devices.

Here, we report the effect of the oxidation pathway on the reversibility of the topotactic transformation between brownmillerite, BM, (Ca,Sr)FeO_2.5_ and perovskite, PV, (Ca,Sr)FeO_3.0_. We have used gas (O_2_/O_3_), liquid (NaOCl in H_2_O), and solid electrolyte ZrO_2_:Y_2_O_3_ (YSZ) for oxygen insertion into the BM phase. These routes explore very different mechanisms of oxygen exchange: the dissociation and reduction of surface-adsorbed O_2_ molecules by direct electron transfer from Fe(III/IV), plus diffusion of O^2–^ into the oxide lattice; competing redox reactions with ^–^OCl and HO^–^ involving lattice oxygen evolution; and direct injection of O^2–^ by an electric field across the solid–solid interface between the oxide film and the YSZ electrolyte.

We show that negative charge-transfer oxides are intrinsically unstable under wet conditions, which lead to important microstructural and compositional changes and a lack of structural/chemical reversibility during oxidation/reduction cycles. Repeated oxidation in O_2_/O_3_ at high temperatures also accumulates lattice defects that produces a progressive loss of cyclability.

On the other hand, electric-field-assisted exchange of oxide ions across the (Ca,Sr)FeO_*x*_/YSZ interface results in full structural and thermal conductivity cyclability. Our work shows the limitations of topochemical oxygen intercalation and discusses the best routes for the development of viable oxide devices based on reversible O^2–^ exchange.

## Experimental Section

Thin films of CaFeO_*x*_ and SrFeO_*x*_ were deposited on (0 0 1) SrTiO_3_ substrates by pulsed laser deposition under identical conditions of temperature, 675 °C, oxygen pressure, 100 mTorr, and laser fluence, ≈1.5 J/cm^2^. Wet oxidation was performed by soaking thin films with the brownmillerite structure in a solution of NaOCl (14% of active chlorine, diluted at 50% with water) for 2 h at 80 °C. For SrFeO_*x*_, gas-phase oxidation was achieved by thermal annealing at 600 °C, 2 h, in 300 mTorr of oxygen, while in the case of CaFeO_*x*_, oxidation was done by thermal annealing under ozone (250 °C, 1 h). The brownmillerite phase was recovered, in all cases, by thermal annealing of the samples at 600 °C, 2 h, at a pressure of 10^–6^ Torr.

For solid-state oxygen exchange experiments, thin films of CaFeO_*x*_ and SrFeO_*x*_ were deposited on (0 0 1) YSZ, with an intermediate layer of CeO_2_ (≈10 nm) at 650 °C and 1 mTorr of oxygen. Oxygen intercalation/deintercalation was done by applying positive/negative voltage to the (Ca/Sr)FeO_*x*_/CeO_2_/YSZ structure using Al and Pt as top and bottom contacts, respectively.

## Results and Discussion

### Why SrFeO_*x*_ and CaFeO_*x*_?

Achieving a *M*^4+^ oxidation state for a 3d ion in a *A*^2+^*M*O_3_ perovskite becomes progressively difficult as the atomic number increases: the σ* band derived from the metal–oxygen antibonding interaction may cross the nonbonding O 2p states so that a *M*^3+^*L* (*L* meaning a ligand-hole), rather than a pure *M*^4+^ ground state, is stabilized.^18^ These are the so-called negative charge-transfer oxides, in which Δ = [*E*(*M*^3+^*L*) -*E*(*M*^4+^)] < 0.^19,20^ Placing the Fermi energy at the O 2p band reduces the enthalpy of oxygen vacancy formation and the oxygen surface exchange rate, which should facilitate the reversible deintercalation of O^2–^-ions between the BM and the PV.^21^

SrFeO_3_ shows a Δ ≈ 0,^20,22^ but still a very small oxygen vacancy formation energy and a high oxygen surface exchange rate and mobility.^21,23,24^ Replacing Sr by Ca reduces the Fe–O–Fe bond angle from 180° in SrFeO_3_ to 180° – ϕ = 158° in CaFeO_3_,^25^ which, according to tight binding theory, reduces the bandwidth proportional to cos(ϕ) ≈ 0.92, and makes Δ more negative. This small difference is enough to make BM (*x* = 2.5) and oxygen-deficient PV (*x* = 3 – δ) the thermodynamically stable phases for CaFeO_*x*_ and SrFeO_*x*_, respectively (Figure S1).

Moreover, ab initio calculations confirm that the thermal conductivity of PV CaFeO_3_ is considerably larger than the BM and shows a strong thickness dependence (see Figure S2 and details of the calculation in the Supporting Information).^26−28^

Therefore, CaFeO_*x*_ and SrFeO_*x*_ provide an opportunity to compare the reversibility of the thermal conductivity after BM ⇆ PV transformation via reversible oxygen insertion in two negative charge-transfer oxides with very similar structural and chemical composition but with a different distribution of holes between the O 2p and *M*^4+/3+^- electronic band of states.

For this study, epitaxial thin films of CaFeO_*x*_ (CFO) and SrFeO_*x*_ (SFO) were deposited on (0 0 1)-STO by PLD, under identical conditions of temperature, 675 °C, and oxygen pressure, 100 mTorr. Thermal conductivity was measured by Frequency Domain Thermoreflectance (FDTR;^29−31^ see Supporting Information for further details of the method).

### Gas-Phase O_2_/O_3_ and Liquid-Phase NaOCl/H_2_O Oxidation

**Figure 1:**
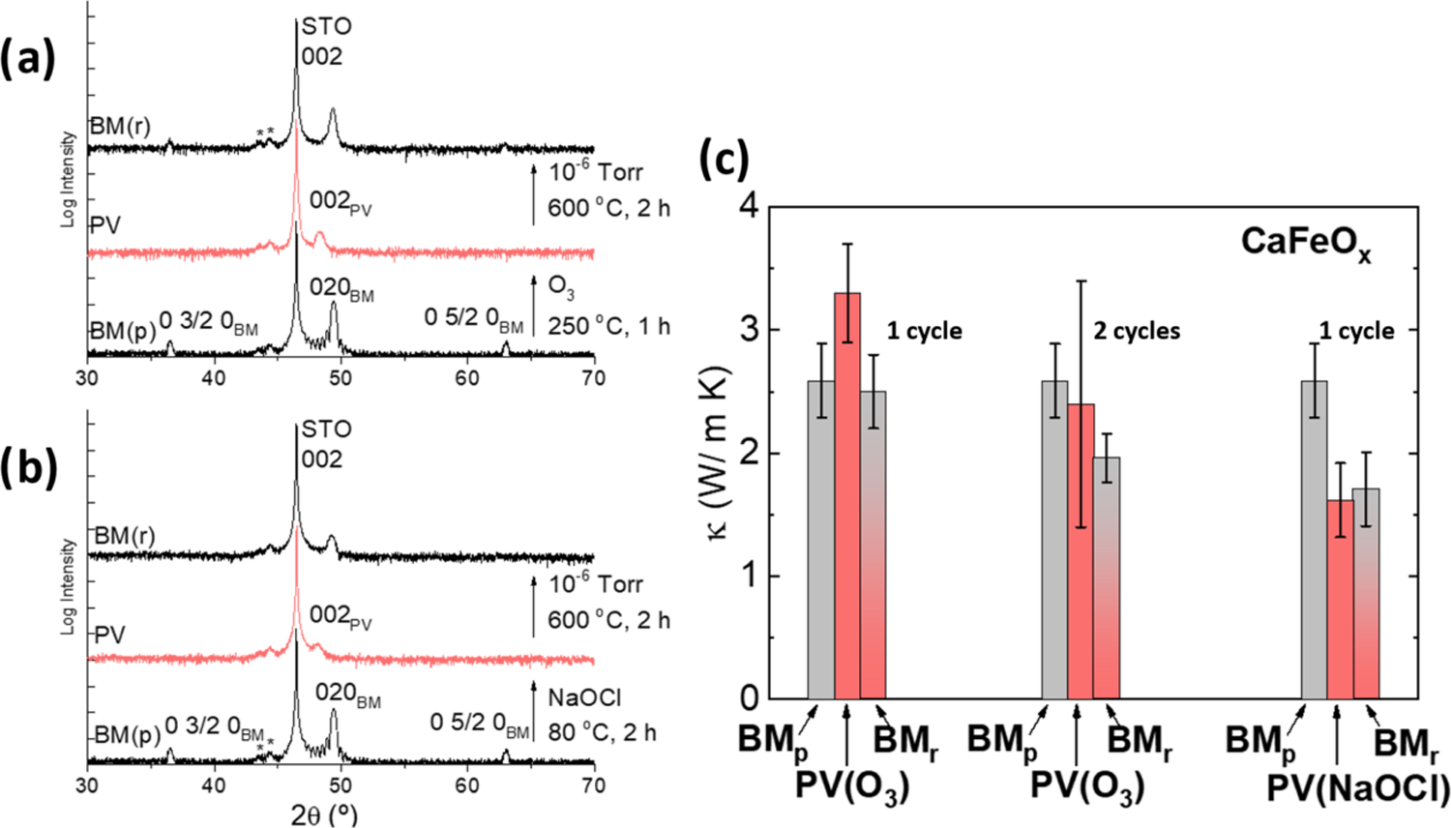
Structural and thermal conductivity reversibility of CaFeO_*x*_. X-ray diffraction patterns of the BM → PV → BM transformation in CaFeO_*x*_ thin films (≈40 nm) using gas O_3_ (a), and liquid NaOCl/H_2_O solution (b). BM_p_ and BM_r_ refer to the pristine and reduced BM phases, respectively. The two peaks at 45° marked with an asterisk are reflections from tungsten of the X-ray tube. The (0 5/2 0) peak of the BM is barely visible after one oxidation cycle with O_3_, but it is completely absent after oxidation with NaOCl/H_2_O. The intensity of the (0 0 2) peak is also reduced in the PV obtained with NaOCl/H_2_O compared to that of O_3_. (c) Thermal conductivity of CaFeO_*x*_ thin films after the BM → PV→ BM transformation, under O_3_ (after the first and second oxidation/reduction cycle) and wet NaOCl/H_2_O. Although the thermal conductivity is reversible after one oxidation/reduction cycle with ozone, repeating the process results in the progressive accumulation of defects and incomplete transformation, leading to a reduction of thermal conductivity. On the other hand, oxidation in NaOCl/H_2_O produces a severe loss of crystallinity and irreversibility of the thermal conductivity, already after the first cycle.

In Figure 1a, we show the X-ray diffraction patterns of CFO after BM → PV→ BM transformations using O_3_ as an oxidant. Annealing the PV under vacuum recovers the BM phase, although the X-ray diffraction reveals the loss of the Laue oscillations around the (0 2 0) peak and the reduction of intensity of the half-order reflections (0 3/2 0) and (0 5/2 0) with respect to the pristine phase, indicating a loss of crystalline quality after just one cycle of topotactic oxygen exchange.

Still, oxidizing the BM to PV with ozone increases the thermal conductivity ≈30%, in very good agreement with the theoretical calculation for 40 nm films, see Figures 1c and S2. However, repeating the oxidation/reduction cycle results in the accumulation of structural defects and incomplete structural transformation, which is reflected in a large variability and irreproducibility of the thermal conductivity upon cycling; see Figures 1c and S3.

On the other hand, wet oxidation of BM CFO with NaOCl/H_2_O also transforms the BM into the PV phase,^32^ although the intensity of the diffraction peaks decreases considerably compared to the case of O_3_ (Figure 1b). Subsequent thermal annealing under vacuum recovers the main (0 2 0) reflection of the BM, but also with a reduced intensity and displacement to lower angles (increased *c*-axis unit cell parameter). Moreover, the loss of half-order reflections in the reduced BM indicates the partial loss of structural long-range order with respect to the pristine sample (see also Figure S4).

The thermal conductivity of the PV obtained by wet chemical oxidation is actually reduced by ≈35% with respect to the original BM (Figure 1c). More importantly, this is an irreversible process: the thermal conductivity does not recover after a subsequent reduction to BM.

**Figure 2:**
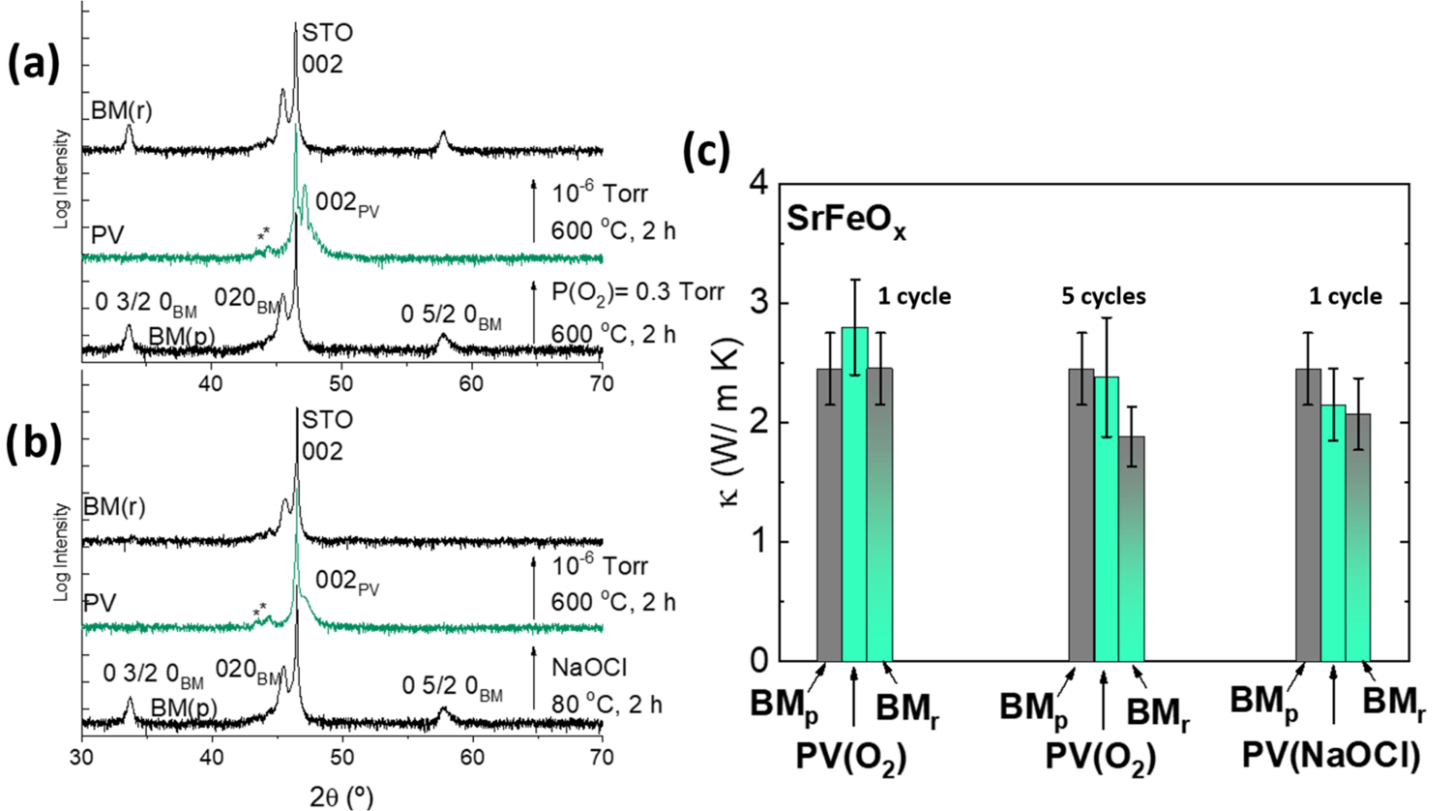
Structural and thermal conductivity reversibility of SrFeO_*x*_. X-ray diffraction patterns of the BM → PV→ BM transformation in SrFeO_*x*_ thin films (≈40 nm thick) using gas O_2_ (a), and NaOCl/H_2_O solutions (b). The two peaks at 45° marked with an asterisk are reflections from tungsten of the X-ray tube. (c) Thermal conductivity of SrFeO_*x*_ thin films after the BM → PV→ BM transformation, with O_2_ (after 1 and 5 cycles) and NaOCl/H_2_O. The thermal conductivity seems reversible after just one cycle of oxidation with O_2_, but the progressive accumulation of defects after further cycling leads to a substantial decrease of thermal conductivity and to its irreversibility upon reduction/oxidation cycles. On the other hand, the process is completely irreversible from the first oxidation cycle with NaOCl/H_2_O.

The results are qualitatively similar in thin films of SrFeO_*x*_ of comparable thickness (Figure 2). In this case, thermal annealing under flowing O_2_ is enough to form the PV, which shows a larger thermal conductivity than the BM. Thermal annealing under vacuum recovers the BM phase and the value of the thermal conductivity, within error, confirming the reversibility of the process after one oxidation/reduction cycle. However, cycling the samples 5 times results in a decrease of κ, between 15–30% with respect to the first cycle (Figure 2c). This, again, points toward a progressive accumulation of defects during topochemical oxidation with gas and poses an important limitation to the design of tunable thermal (and probably of other type) devices based on this oxidation route.

On the other hand, as for CFO, the X-ray diffraction pattern of the PV obtained after wet oxidation in NaOCl/H_2_O also shows the loss of crystallinity, reflected in the decrease of intensity and broadening of the diffraction peaks (Figure 2b). This also results in the irreversible reduction of the thermal conductivity, already after the first oxidation cycle (Figures 2b,c and S4).

**Figure 3:**
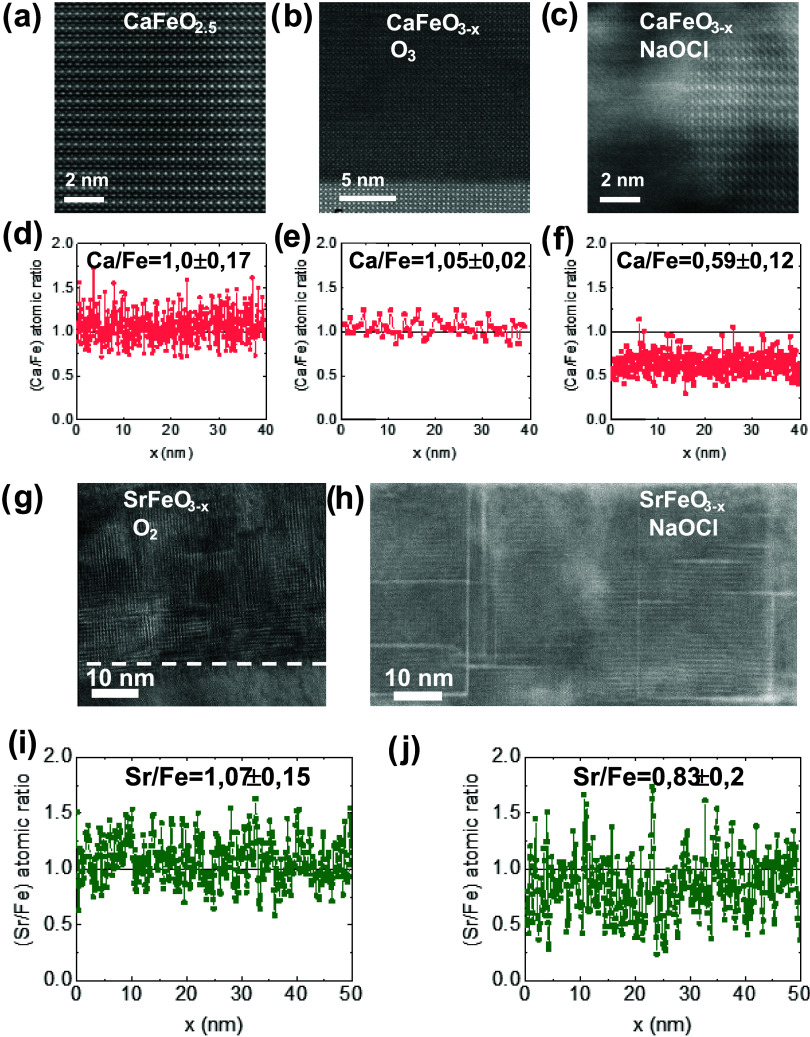
Microstructure and chemical compositions of CaFeO_*x*_ and SrFeO_*x*_. High-resolution HAADF-STEM images of the BM thin film of CaFeO_2.5_ (a), and the PV obtained by oxidation with O_3_ (b) and NaOCl (c). Wet oxidation in NaOCl/H_2_O induces the formation of amorphous regions, which coexist with crystalline domains of the PV structure. The corresponding EDS chemical profile analysis along the films are shown under each image (d–f). The Ca/Fe ratio decreases after oxidation of the BM to PV with NaOCl/H_2_O, confirming the partial dissolution of Ca^2+^. The crystalline structure of PV SrFeO_3_ obtained after thermal annealing in pure O_2_ and by wet oxidation in NaOCl/H_2_O is shown in the TEM (g) and BF-STEM (h) images, respectively. The mosaic structure of nanodomains with different orientation is appreciated in (g), while clear signs of amorphization along the straight paths of grain boundaries are appreciated in (h). The EDS chemical profile analysis along the films shows Sr/Fe ≈1.07 ± 0.15 for the sample oxidized in O_2_ (i), and a decrease in the Sr^2+^ content, Sr/Fe ≈0.83 ± 0.2, for the samples oxidized in NaOCl/H_2_O (j).

For a detailed understanding of the effect of gas O_2_/O_3_ and liquid NaOCl/H_2_O topochemical oxygen insertion on the microstructure of the oxides, we performed high-resolution scanning transmission electron microscopy (STEM). Cross-sectional lamellae after BM-PV cycles with both types of oxidants were analyzed by high-angle annular dark field imaging (HAADF) and energy dispersive X-ray spectroscopy (EDS) in STEM. These results are summarized in Figure 3.

Pristine BM CaFeO_2.5_ shows good crystallinity, and an atomic ratio Ca/Fe ≈1; oxidation with O_3_ maintains the Ca/Fe ≈1 and the crystal structure (see also Figure S5). However, wet oxidation in NaOCl/H_2_O results in partial amorphization of the film, which coexist with crystalline domains (Figure 3c). Also, the Ca/Fe ratio decreased to ≈0.59, indicating substantial Ca^2+^ leaching to the solution. Increasing the concentration of oxidant results in complete amorphization of the film after a few minutes and low atomic Ca/Fe ratio (see Figure S6).

Similar results are found in SFO: oxidation of the BM under flowing O_2_ maintains the good crystallinity and the stoichiometric atomic ratio Sr/Fe ≈1.07 ± 0.19 (Figure 3g,i); obtaining the PV under wet oxidation conditions results in partial amorphization, although in this case the amorphous regions are more restricted to the boundaries between crystalline nanodomains (Figure 3h). The higher stability of SFO compared to CFO is also manifested in an atomic ratio Sr/Fe ≈ 0.83 ± 0.23, suggesting a smaller dissolution rate of Sr^2+^.

The structural/chemical differences observed between the PV obtained by gas and wet oxidation can be understood from the details of the mechanism responsible for the incorporation/release of the O^2–^ in negative charge-transfer oxides. Topochemical oxidation with O_2_/O_3_ starts with the absorption of the oxidant molecule on the surface, and the electron transfer from Fe^3+^ to the antibonding molecular orbitals of the O_2_/O_3_. This weakens the O–O bond and forms O^2–^ ions, which diffuse into the structure and fill the oxygen vacancies of the BM, forming the PV. Reducing the sample back to the BM implies the dimerization of O^2–^ ions into peroxide O_2_^2–^ or superoxide O_2_^–^ species inside of the lattice prior to leaving the material as O_2_, which leads to a decrease in the O-(Ca/Sr) and O–Fe bonding. This, combined with a moderate ionic mobility at the temperature of the experiments (600 °C), may lead to irreversible cation segregation or accumulation of lattice defects, as observed in other oxides after repeated ion intercalation cycles.^15,16^ This is the most probable cause of the progressive decrease in thermal conductivity upon repeated cycling.

In the case of NaOCl/H_2_O, the strong oxidation potential of ^–^OCl ions is large enough to oxidize Fe^3+^ in (Ca,Sr)FeO_2.5_ to a predominant Fe^3+^*L* electronic configuration in (Ca,Sr)FeO_3–*x*_, which catalyze the evolution of O_2_ (OER) in water, with the important participation of lattice oxygen in the process.^33−35^ Therefore, during the oxidation of Fe^3+^ by NaOCl in water, there is a competition between the incorporation of O^2–^ from the hypochlorite to the oxide lattice and the formation of oxygen vacancies associated with the OER cycle. The rapid formation of oxygen vacancies by the second mechanism, combined with the high solubility of Sr^2+^ and Ca^2+^ ions, may result in the collapse and amorphization of the whole structure.^33,36−39^

**Figure 4:**
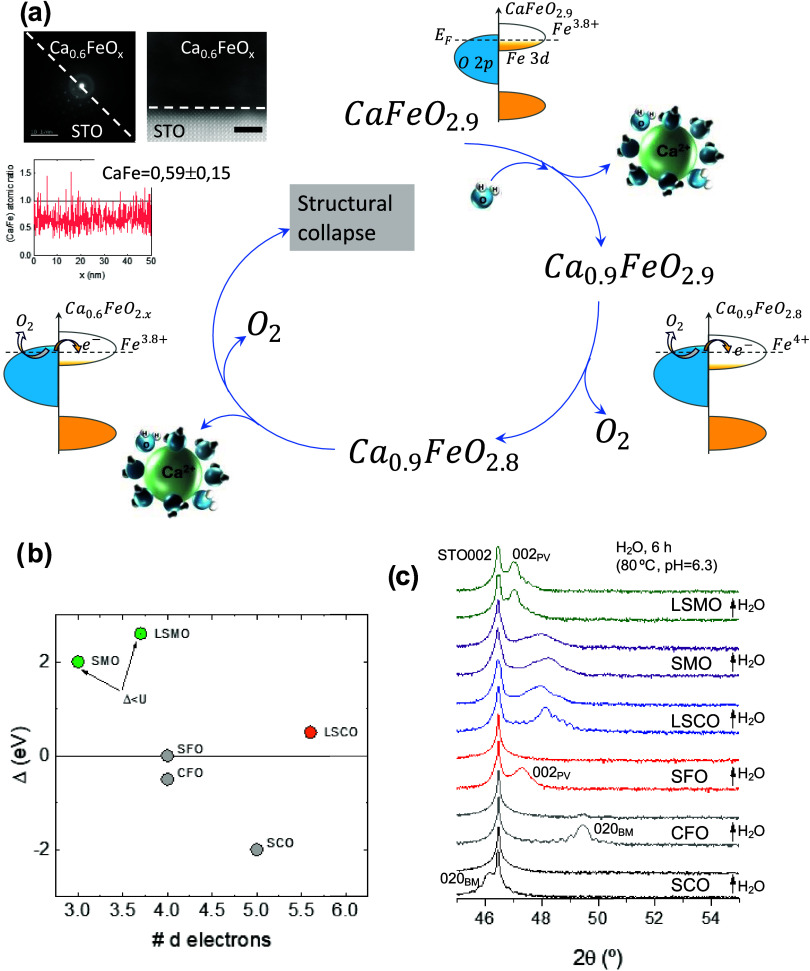
Stability of negative charge-transfer oxides in water. (a) Proposed feedback mechanism for amorphization of Δ < 0 materials, exemplified for CaFeO_*x*_. Intrinsic oxygen vacancies reduce the coordination of surface Ca^2+^, which promotes its lixiviation. This, in turn, increases the oxidation state of Fe to keep the electrical neutrality, which results in further electron transfer from the O 2p band and O^2–^ to O_2_ oxidation, as shown in the corresponding band diagrams. The vicious cycle of Ca^2+^ dissolution/O^2–^ oxidation continues until the full collapse of the structure. The pictures in the left-top corner show the STEM image (scale bar 2 nm) of an amorphous film with a Ca/Fe ≈ 0.6 and the corresponding FFT. The absence of discrete Bragg spots in the portion of the film confirms the complete structural amorphization after immersion in deionized hot water for 6 h. In part (b), we show the values of Δ *v*s number of d electrons for the oxides studied in this work. Gray, orange, and green correspond to negative charge-transfer, positive charge-transfer, and Mott–Hubbard oxides, respectively. (c) Comparison of the X-ray diffraction patterns (intensity in logarithmic scale) of the oxides in (b) before and after immersion in deionized water at 80 °C for 6 h. The pristine oxides show the amorphization of the structure, reflected in the loss of the diffraction peaks of the film, which becomes more evident as Δ becomes more negative.

Actually, if this mechanism is correct, it should lead to the instability of negative charge-transfer oxides in pure water without the need for any chemical or electrochemical oxidant. Having Δ < 0 implies the presence of a given amount of intrinsic O^2–^ vacancies to equilibrate the O_2_/O^2–^ and Fe^4+^/Fe^3+^ redox pairs. When this concentration of oxygen vacancies is large enough, the coordination of Ca^2+^/Sr^2+^ decreases to a point that, along with the large solubility enthalpy of alkaline-earth cations, results in their partial leaching from the surface to the solution. This, in turn, will increase the oxidation state of Fe, promoting further electron transfer from the O 2p band and O^2–^ oxidation to O_2_, which reduces further the coordination of the alkaline-earth cation and promotes their dissolution. This vicious cycle will continue until the total collapse of the structure, as schematically shown in Figure 4a. We want to emphasize, again, that this mechanism does not require the participation of a chemical or electrochemical oxidation; it is the combined effect of Δ < 0 plus the large solubility of Ca^2+^/Sr^2+^ that drives this spontaneous instability in water.

To probe this hypothesis, we have studied the stability in pure water of a series of oxides with an increasing relevance of their charge-transfer energy, Δ. Thin films of SrCoO_3_ (SCO), CFO, SFO, La_0.6_Sr_0.4_CoO_3_ (LSCO), SrMnO_3_ (SMO), and La_0.7_Sr_0.3_MnO_3_ (LSMO), of comparable thickness ≈40 nm, were immersed in deionized hot water at 80 °C, pH = 6.3, for 6 h. The value of Δ increases along this series (see Figure 4b) from negative in SCO to positive in LSCO. For LSMO and SMO, Δ is smaller than the Mott–Hubbard energy (*U*), so that the lattice oxygen is not a redox-active species. In this case, an initial dissolution of a small amount of Sr^2+^ will be compensated by an increase in the oxidation state of the metal, instead of O^2–^→ O_2_, ending the feedback loop of oxygen vacancies-ion leaching.

As shown in Figure 4c, SCO, CFO, and SFO (all Δ < 0) become amorphous after 6 h in the hot water. However, LSCO (Δ > 0) only shows a reduction of intensity and broadening of the diffraction peaks, signaling a partial loss of crystallinity (probably on the surface). Finally, the Mott–Hubbard oxides LSMO and SMO (Δ < *U*) remain practically unaffected by immersion in hot water.

This confirms the important role of lattice oxygen in the spontaneous amorphization of negative charge-transfer oxides.

Surface corrosion was reported in films of La_0.5_Sr_0.5_CoO_3_ and SrCoO_3_ during topochemical BM ⇆ PV transformation with ionic gels, which prevented the full reversibility of the transformation.^10,17^ This behavior was attributed to acid etching from a large concentration of H^+^, generated from electrochemical splitting of residual amounts of H_2_O in the gel. However, the anions of the ionic liquid gel will also coordinate with alkaline-earth cations, facilitating the dissolution of the film by the mechanism proposed in Figure 4a. Therefore, the inherent instability of negative charge-transfer oxides impedes their BM ⇆ PV cyclability under wet conditions (either in water or other polar ionic liquids or gels), so these routes should be avoided for the fabrication of functional tunable devices.

The discussion of the results in Figure 4 have also their relevance in the interpretation of the surface reconstruction observed in Fe and Co oxide catalysts under OER conditions.^33,39,40^

### Solid-State Oxygen Exchange with the Y_2_O_3_:ZrO_2_ (YSZ) Electrolyte

Finally, we discuss the reversible intercalation of oxygen into CaFeO_*x*_ and SrFeO_*x*_ by using YSZ as a solid electrolyte. YSZ is an oxide-ion conductor, which has been previously used as an oxygen reservoir to exchange O^2–^ ions in Co-oxides.^41−44^ In this case, we probed the reversibility of the BM ⇆ PV transformation by direct injection of O^2–^ ions across the YSZ/(CFO, SFO) interface with an electric field.

**Figure 5:**
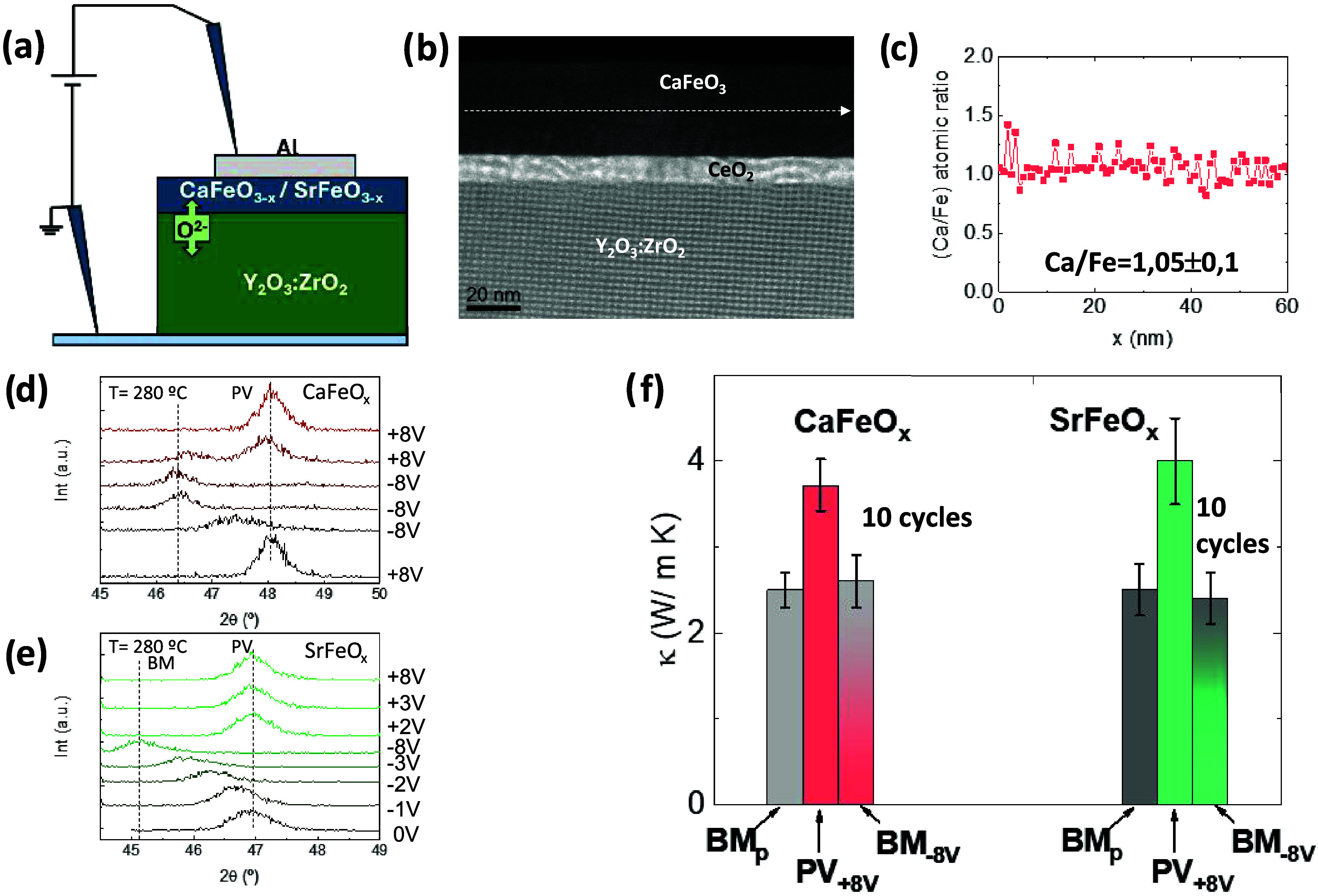
Reversibility of thermal conductivity after solid-state topotactic oxygen insertion. (a) Scheme of the Pt/YSZ/(Ca,Sr)FeO_*x*_/Al for O^2–^ insertion with an electric field. (b) HAADF-STEM image of a film of CaFeO_*x*_ on CeO_2_/YSZ. In panel (c), we show the EDS line scan along the film, after several oxidation/reduction cycles, and final oxidation at +8 V to the perovskite phase; the film maintains the stoichiometric ratio Ca/Fe = 1. (d, e) In-operando X-ray diffraction patterns of the BM → PV → BM transformation in CaFeO_*x*_, and SrFeO_*x*_ thin films (≈140 nm) at 280 °C, under different applied voltages. The films can be cycled between both structures dozens of times without apparent damage. Intermediate phases (or coexistence of phases) can be stabilized by using intermediate voltages between the extreme ±8 V. The diffractograms in panels (d) and (e) correspond to consecutive experiments; each diffractogram takes 8 min to complete. (f) Reversibility of the thermal conductivity in the films of (Ca,Sr)FeO_*x*_ after topochemical oxygen insertion from YSZ. The samples have been cycled at least 10 times before taking the measurements. No degradation of the thermal conductivity was observed after repeated cycling of the samples.

For that, we deposited epitaxial thin films of CaFeO_2.5_ and SrFeO_3–*x*_ on (0 0 1) YSZ, with an intermediate layer of CeO_2_ (≈10 nm) to prevent the formation of (Ca,Sr)ZrO_*x*_, which blocks the oxygen migration (Figure 5b). Due to the low thermal conductivity of the YSZ substrates, several thin films of CFO and SFO within a thickness range of 30 to 140 nm were prepared for increasing the accuracy of the thermal conductivity analysis.

For oxygen intercalation into the BM film, a positive voltage was applied to the (Ca,Sr)FeO_*x*_/CeO_2_/YSZ structure, using Al and Pt as top and bottom contacts, respectively, at the time that the transformation was being followed by in-operando X-ray diffraction at 280 °C (see Supporting Information for further details of the experiment). The results are shown in Figure 5d,e.

The electrochemical process takes place according to the following equilibrium:^45^
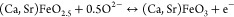
1The electron density injected in the film is given by *Q* = (*I·t*)/(*e·V*), where *I*, *t*, *e*, and *V* refer to the current intensity flowing through the sample, the application time of the voltage, the electron charge, and the volume of the film, respectively. According to eq 1, the complete transformation of the BM into PV requires an electron density of around 1.75 × 10^22^ e/cm^3^, and therefore, it can be controlled by the time of during which the voltage is applied through the film (see Figure S7).

In the case of SFO, application of ≈ ± 8 V to the 5 × 5 mm^2^ film on a YSZ substrate 0.5 mm thick at 280 °C results in the stabilization of the current and change of color (see Figure S7) after ≈1.5 min, for 140 nm thick films. Smaller voltages were applied during different times to achieve different oxidation states.

The diffusion coefficient of oxide ions in perovskite thin films is of the order of *D* ≈ 5 × 10^–17^ cm^2^/s at room temperature,^46^ and remains ≈10^–16^ cm^2^/s below 500 °C, so this could be taken as a high limit at the temperature of the experiment of 280 °C.

In this case, the ionic mobility is , which at 8 V would correspond to an equivalent diffusion coefficient of . In this case, the diffusion time along the film will decrease the dozens of seconds, in the same order observed in our films.

The velocity of the PV→ BM transformation can be reduced by reducing the electric field (Figure 5e), observing a continuous evolution of the lattice parameter during the transformation (Figure S7). On the other hand, the transformation back to the PV occurs much faster, even at ≈2 V, so no intermediate phases can be stabilized during the time of the X-ray diffraction measurement (8 min). From the total amount of charge transferred, the estimated oxygen content in the PV is close to 3.0 (Figure S7).

In the case of CFO, the transformation from PV-to-BM is slower and takes several minutes to complete even at −8 V. However, as in SFO, it occurs much faster in the BM-to-PV direction.

Postiglione et al.^17^ reported a detailed study of the kinetics of the BM ⇆ PV transformation in ultrathin films of La_0.5_Sr_0.5_CoO_3_ by ion-gel gating. Contrary to our observation, they found that the oxidation BM → PV is slower than the reduction PV → BM. Given the similar crystal structures, we do not expect a large difference in the oxygen diffusion coefficient of these Co and Fe oxides. However, the mechanism of oxidation is fundamentally different in both methods: while it requires the electrochemical formation of the oxidative species (O^2–^, O^–^, etc.) and their diffusion from the surface to the interior of the film in the ion-gel method, the use of a solid electrolyte produce the direct injection of O^2–^ ions across the oxide/YSZ interface. This is an important mechanistic difference that could be the reason for the discrepancy observed between both methods. In any case, this fact deserves further investigation. Faster X-ray diffraction experiments in a synchrotron could provide valuable information about the nature of the phase transition in both directions.

We have repeated the experiments and corroborated that the structure of the films can be switched between BM and PV at least 10 times with similar results and without apparent degradation. EDS analysis after repeated switching also shows that the Ca/Fe ratio remains ≈1 (Figure 5c).

Regarding the thermal conductivity, it increases 50–60% in the PV with respect to the BM, in both CaFeO_*x*_ and SrFeO_*x*_ (Figure 5f). More importantly, the large contrast in the thermal conductivity is maintained after cycling the samples BM ⇆ PV several times. We repeated the experiments with different samples of thicknesses from 30 to 140 nm, with comparable results.

It is important to note that the electrical resistance of the films varies between 3–50 mΩ cm^–1^ at room temperature, depending on the thickness and composition. This accounts for a maximum contribution to the thermal conductivity between ≈0.01 and 0.2 W m^–1^ K^–1^, which confirms that the change of κ observed in the paper cannot be due to an electronic contribution but to a structural change.

Therefore, the exchange of O^2–^ ions across the (Ca,Sr)FeO_*x*_/YSZ solid interface presents the best option in terms of topochemical reversibility in negative charge-transfer oxides. The absence of other competing reactions, the avoidance of molecular O_2_ bubbles forming inside the structure during oxygen deintercalation, and the use of relatively low temperatures avoids the degradation of the structure and maintains the physical properties of the oxide upon repeated cycling.

## Conclusions

Our study demonstrates that reversible and repeatable manipulation of physical properties of functional oxides through topochemical oxygen exchange depends very much on the oxidation pathway. Negative charge-transfer oxides are intrinsically unstable in H_2_O (and probably in other polar and ionic solvents), which limits the applicability of wet methods for the fabrication of tunable devices based on these materials. This instability reflects the active role played by O 2p states as redox species in negative charge-transfer oxides. Repeated cycles of oxidation/reduction using gas O_2_/O_3_ oxidants also lead to a progressive accumulation of defects, which limits the full structural reversibility, which is reflected in the lack of reversibility of the thermal conductivity.

On the other hand, the direct exchange of O^2–^ ions with solid electrolytes allows a truly reversible tuning of the structure and physical properties, showing that this is the milder method of topotactic oxygen exchange.

The results presented in this paper should be taken into consideration when proposing topotactic oxygen exchange as the working principle of tunable oxide devices.

## Data Availability

The authors declare that all data supporting the findings of this study are available within the article and its Supporting Information Files. Additional experimental and computational data are available from the corresponding author, on reasonable request.

## Supporting Information Available

Additional information about the preparation and structural characterization of the samples, as well as about the thermal conductivity measurements and analysis (PDF)
