# Supplementary material for: Exploring Topochemical Oxidation Reactions for Reversible Tuning of Thermal Conductivity in Perovskite Fe Oxides
Source: Chem Mater. 2024 Oct 9;36(20):10249–58. doi: 10.1021/acs.chemmater.4c02023 (PMC11500631; doi:10.1021/acs.chemmater.4c02023)
Supplement: Supplementary file 1 — cm4c02023_si_001.pdf [file cm4c02023_si_001.pdf]

Supplementary information for:

Exploring topochemical oxidation reactions for reversible tuning of thermal conductivity in perovskite Fe oxides

Noa Varela-Domínguez,[1] Marcel S. Claro,[1] Enrique Carbó-Argibay,[2] César Magén,[3] Francisco Rivadulla.[1,*]

[1] Centro Singular de Investigación en Química Biolóxica e Materiais Moleculares (CIQUS), Departamento de Química-Física, Universidade de Santiago de Compostela, 15782 Santiago de Compostela, Spain.

[2] International Iberian Nanotechnology Laboratory (INL), Avenida Mestre José Veiga s/n, 4715-330 Braga, Portugal.

[3] Instituto de Nanociencia y Materiales de Aragón (INMA), CSIC-Universidad de Zaragoza, 50009 Zaragoza, Spain.

Pulsed laser deposition of the films and reduction/oxidation process.

Thin films of $CaFeO_x$ and $SrFeO_x$ were deposited on (0 0 1)-STO by PLD, under identical conditions of temperature, 675 ºC, oxygen pressure, 100 mTorr, and laser fluence, ≈ 1.5 J/cm^2. Under these conditions, BM $CaFeO_{2.5}$ and PV $SrFeO_{3-x}$ are the most stable phases. Thermal annealing under ozone (250 ºC, 1 h) was used for chemical oxidation of BM $CaFeO_{2.5}$ to PV $CaFeO_{3-x}$. Reduction of the samples from the PV to the BM was done, in all cases, by thermal annealing at 600 ºC, 2 h, at a pressure of 10^{-6} Torr. On the other hand, annealing at 600 ºC in 300 mTorr of oxygen was enough to transform BM $SrFeO_{2.5}$ into the PV phase.

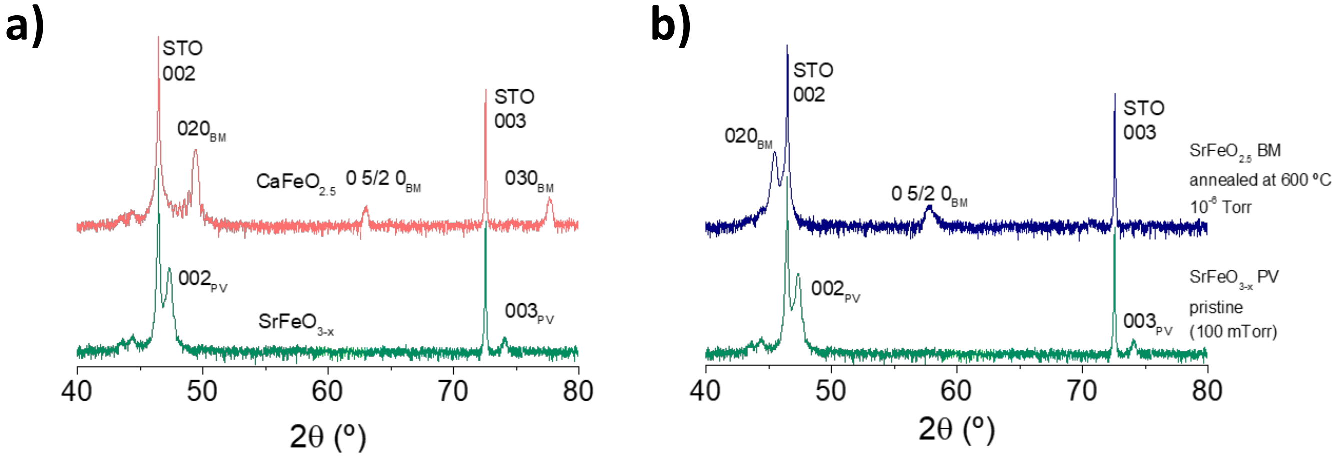

Figure S1. a) X-ray diffraction patterns of PV-SFO and BM-CFO thin films as deposited at 100 mTorr. b) Reduction of the as-deposited PV film of SFO to BM, through thermal annealing under vacuum for 2 h. All films are 40 nm thick.

Ab initio calculations

Phonon band structure and density-of-states, heat capacity, and thermal conductivity of bulk $CaFeO_x$ are calculated by ALAMODE,[1] extracting harmonic and anharmonic interatomic force constants (IFCs) from ab-initio density functional theory calculations with Hubbard correction, DFT+U. The QUANTUM ESPRESSO code, the generalized gradient approximation (GGA) in the Perdew-Burke-Ernzerhof for solids scheme: PBEsol, and the Iron U parameter of 4.0 eV were chosen for all DFT+U calculations, following previous reports of $CaFeO_x$ structure determination.[2] Projected augmented wave pseudopotentials (PAW) from pslibrary[3] were also used. Energy cutoff of 90 Ry, and a charge cutoff of 1080 Ry were selected after convergence tests on those pseudopotentials. The initial structure is obtained from full relaxation of the structure, both atomic positions and lattice parameters, until the atomic force is lower than 0.02 eV/Å. The Brillouin zone was integrated in a 5´5´5 grid for $CaFeO_{2.5}$ and $CaFeO_3$. The second and third-order IFCs were determined by fitting these parameters in DFT+U gamma point calculations of variations of 2´2´2 super-cell where atoms were displaced from equilibrium positions. For second-order IFCs, atomic positions are displaced 0.05%, and for third-order 0.2-0.3%. The total atomic configurations for $CaFeO_{2.5}$ and $CaFeO_3$, were, respectively, 31 and 19 for the second-order and 2195 and 1392 for the third-order IFCs. The fitting error was below 2% for all fittings, and phonon energies are all real (positive), pointing to the stability of simulated structures and the consistency of the method used.[1] From IFCs, thermal conductivity is calculated by ALAMODE solving the Boltzmann Transport Equation (BTE) with relaxation time approximation (RTA). Since the approximation depends on the resolution of phonon reciprocal space, the grid q´q´q was improved until practical computational limitations: q=10 (7 in κ_z direction) for $CaFeO_{2.5}$ and q=9 for $CaFeO_3$. The limit $q\rightarrow\infty$ is obtained by linear extrapolation.

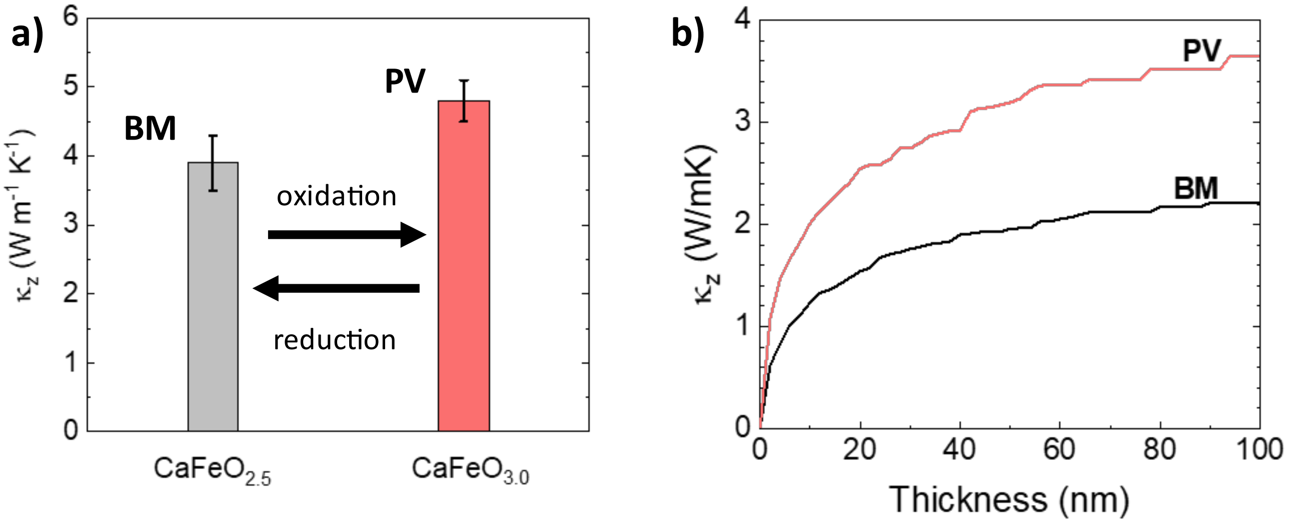

Figure S2. a) ab initio calculated bulk thermal conductivity along the c-axis of brownmillerite (BM) $CaFeO_{2.5}$, and perovskite (PV) $CaFeO_3$. The calculations predict an increase of thermal conductivity of 20 % between bulk BM and PV. Due to the different dependence of κ with thickness (b), larger thermal contrast of ≈40 % can be observed in thin films under 100 nm.

Cyclability of the oxidation/reduction process

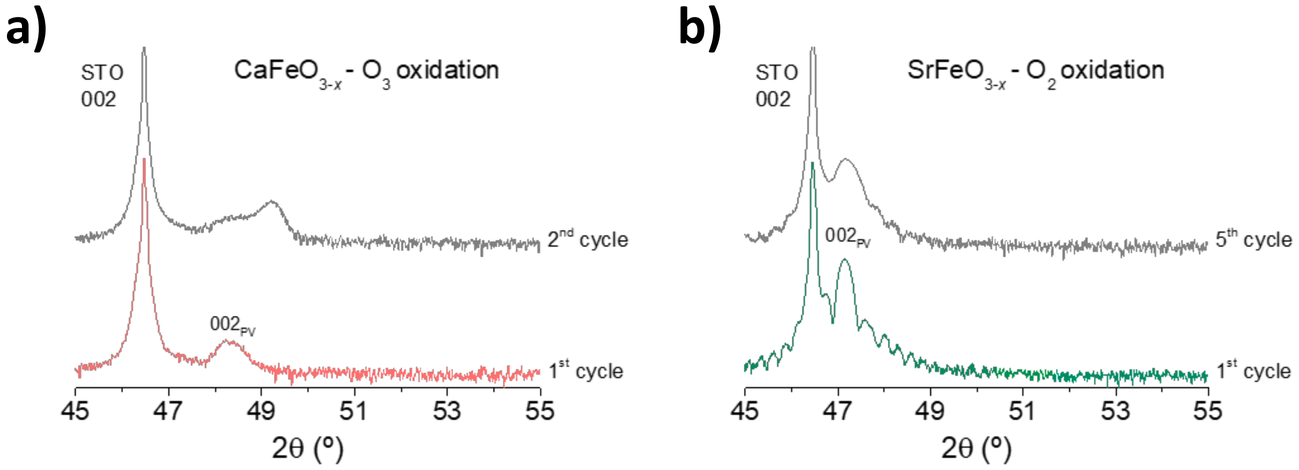

Figure S3. a) Comparison of the X-ray diffraction patterns of the PV $CaFeO_3$ after two oxidation cycles: BM(pristine)→PV(O_3, 1st cycle)→BM(reduced)→PV(O_3, 2nd cycle). After two cycles, there is an incomplete transformation of the BM into PV, showing a mixture of both phases. b) Comparison of the X-ray diffraction patterns of the PV $SrFeO_3$ after five BM⇆PV cycles. The (0 0 2) diffraction peak moves towards lower angles (larger lattice parameter), broadens and losses the Laue oscillations, indicating a reduction of crystalline quality.

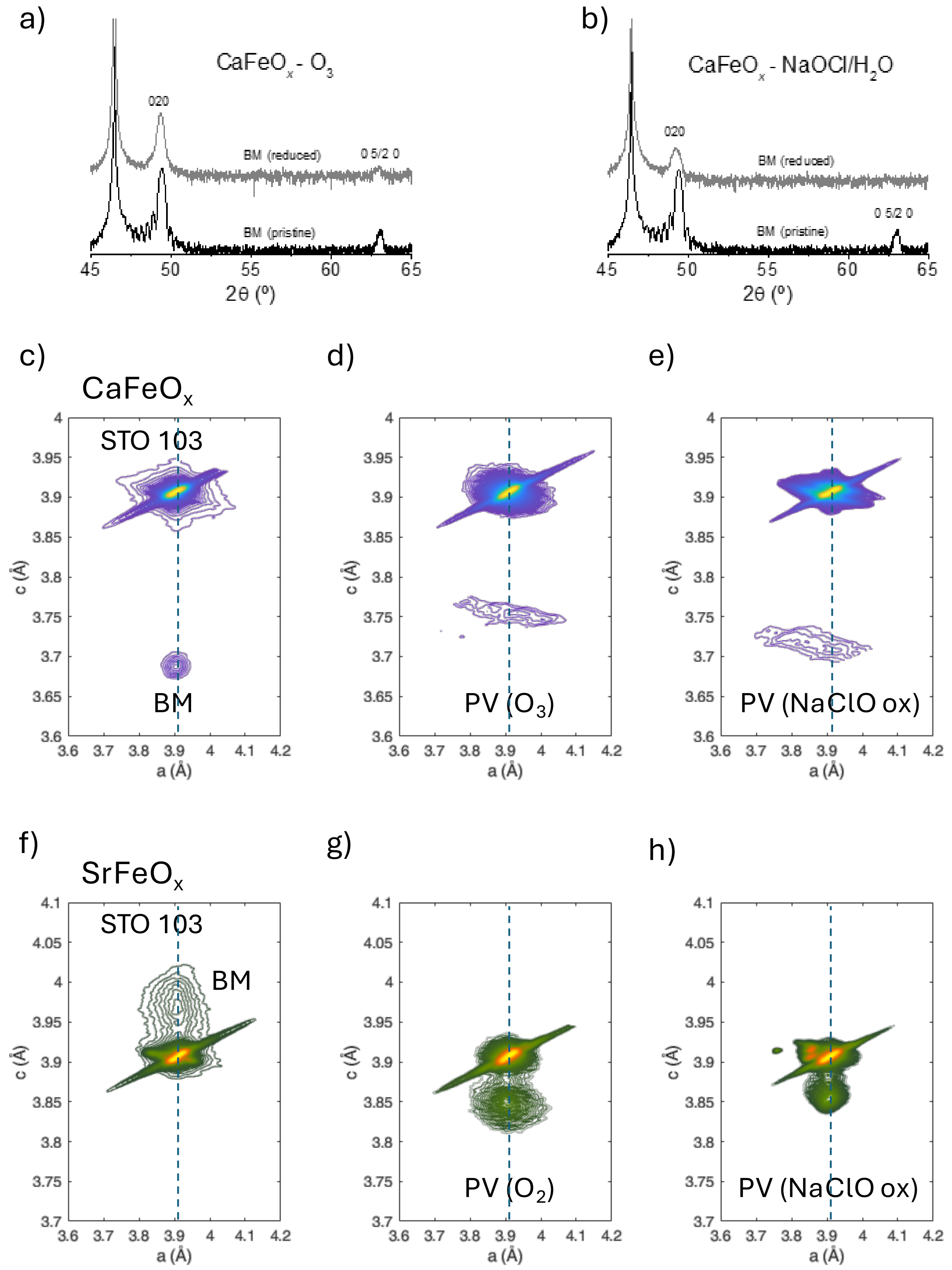

Figure S4. X-ray diffraction patterns of the BM phase of $CaFeO_x$ before and after the topotactic cycle BM→PV→BM, using O_3 (a) and wet NaOCl (b) as an oxidant. The crystallinity of the PV in a) is much better that in b), judging from the higher intensity and narrower (0 2 0) peak, as well as by the presence of the (0 5/2 0),

which is totally absent in the BM derived from the PV oxidized with NaOCl. In c-h) we show the reciprocal space maps around the (1 0 3) reflection of the STO for CFO (c-e) and SFO (f-h). There is a slight relaxation in CFO and the c-axis lattice parameter is shorter in the sample oxidized with NaOCl with respect to ozone. For SFO, the lattice parameters are identical in both cases (NaOCl and oxygen) and the film remains matched to the substrate during the BM to PV transformation.

The PV phase of CFO and SFO can be obtained by soaking films with the BM structure in a solution of NaOCl (14% of active chlorine diluted at 50% with water) for 2 hours at 80 ºC.[4]

In Figure S4 we show a comparison of the BM $CaFeO_{2.5}$ in the pristine state, and after reduction from the PV obtained by O_3 and wet NaOCl/H_2O oxidation, respectively.

The samples for electron microscopy characterization (cross-section lamellae) have been prepared in a DualBeam FIB-SEM FEI Helios G2 NanoLab 450S and were mounted on copper lift-out grids. BF/HAADF-STEM Imaging and EDX spectroscopy characterization were carried out in a FEI Titan G3 Themis Cubed (X-FEG) transmission electron microscope, operated at 200 kV and equipped with Cs DCOR probe corrector and Super-X EDS system. TEM Imaging characterization was performed in a JEOL JEM 2100 (LaB_6) transmission electron microscope, operated at 200 kV and equipped with a Gatan OneView 4k x 4k CMOS camera.

STEM experiments on $CaFeO_3$ grown on STO and YSZ were carried out in an FEI Titan 60-300 Low Base (X-FEG) microscope, operated at 300 kV and equipped with an CETCOR Cs probe corrector from CEOS, and an Ultim Max TLE10 EDS system by Oxford Instruments. In this case, cross-sectional lamellas for STEM analysis were prepared in an FEI Helios NanoLab 650.

The microstructure of the CFO-PV film obtained by oxidation with O_3 is shown in Figure S5. The high-resolution STEM image shows the coexistence of PV and BM domains. However, the X-ray diffraction patterns show the formation of single-phase PV after oxidation with O_3. Thus, the BM domains observed in the image may be caused by partial deoxygenation induced by ion beam irradiation during the FIB preparation of the lamellae for TEM observation.

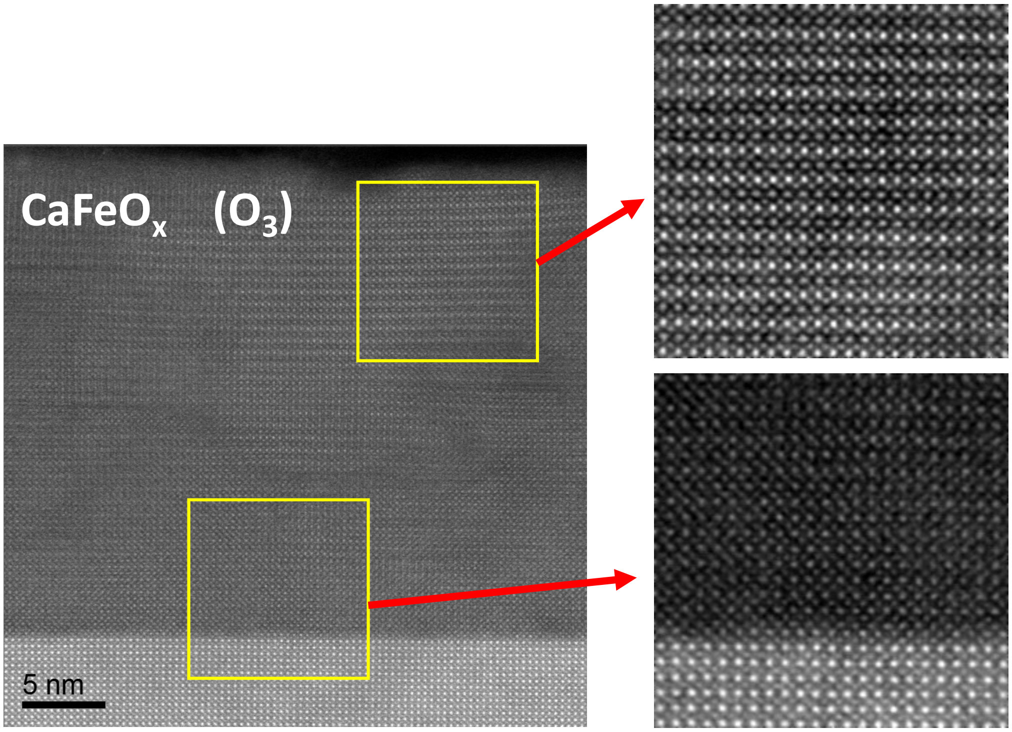

Figure S5. High resolution HAADF-STEM image of a film of $CaFeO_x$ oxidized with O_3. The film shows the coexistence of BM (top) and PV regions (bottom).

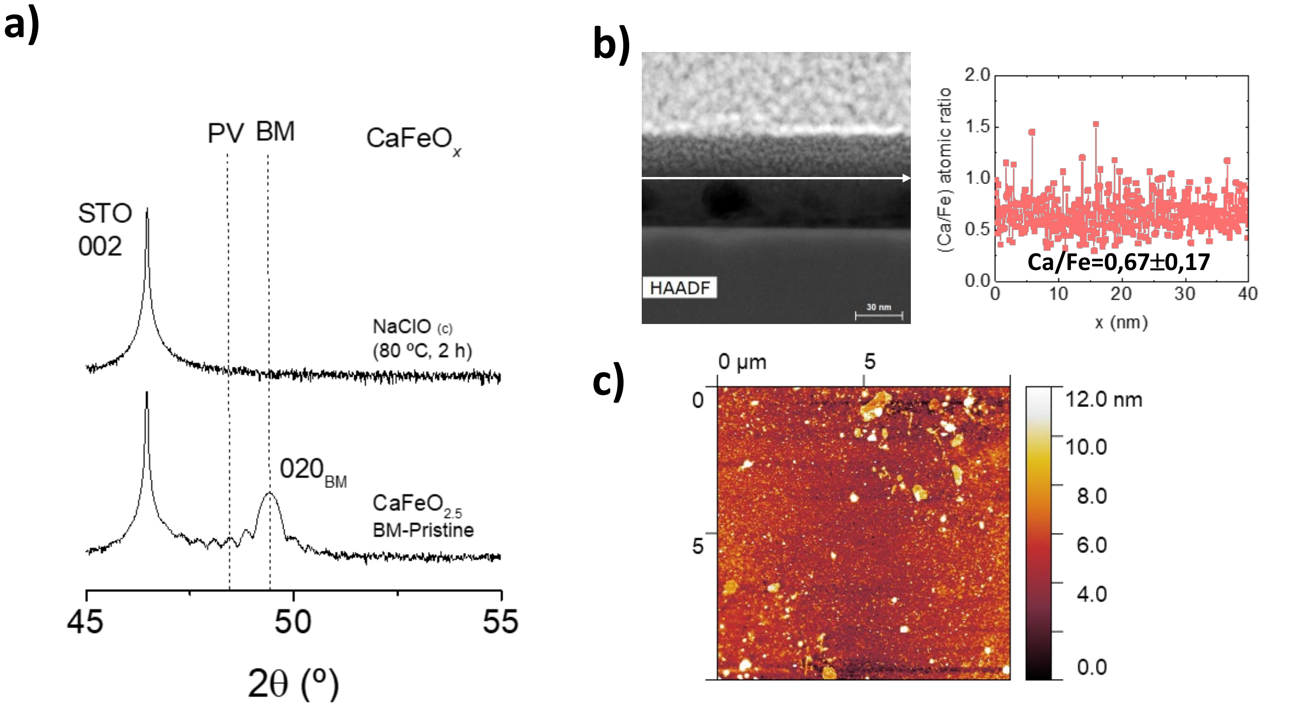

Figure S6. a) X-ray diffraction patterns of BM-CFO films after immersing in hot concentrated solution of NaOCl for 2 h. The disappearance of the Bragg peaks confirms the amorphization of the film. b) HAADF-STEM and EDS analysis of the amorphous film, showing an important reduction of the Ca/Fe content. c) AFM

topography image of a $CaFeO_x$ thin film after treatment with NaOCl in H_2O. The sample surface presents an average roughness of 2.25 nm.

The experimental setup for direct injection of O^{2-} ions from oxide ion conductor Y_2O_3:ZrO_2 into the $CaFeO_{2.5}$ and $SrFeO_{2.5}$ thin films, is shown in Figure 5 a. Repeated BM⇆PV cycles were done in epitaxial thin films of $CaFeO_{2.5}$ and $SrFeO_{2.5}$ on (0 0 1) YSZ, with an intermediate layer of CeO_2 (≈10 nm) with thicknesses between ≈30−140 nm.

During an oxidation experiment, the total amount of charge Q transferred to the film can be obtained by integrating the I/t curves. Given that 2 charges are consumed per oxygen and unit cell, the total amount of charge can be related to the total change in the oxygen content of the film $CaFeO_{2.5+x}$, through:

$$x = \frac{Q}{n_{uc} 2e}$$

Where n_{uc} is the number of unit cells in the whole film, which can be calculated as $n_{uc} = \left(\frac{V_{film}}{V_{uc}}\right)$. The area of our films is 0.5×0.5 mm^2, so that V_{film}=0.5×0.5×t×10^{-6} mm^3, where t is the film thickness in nm. The results are shown in Figure S7 b).

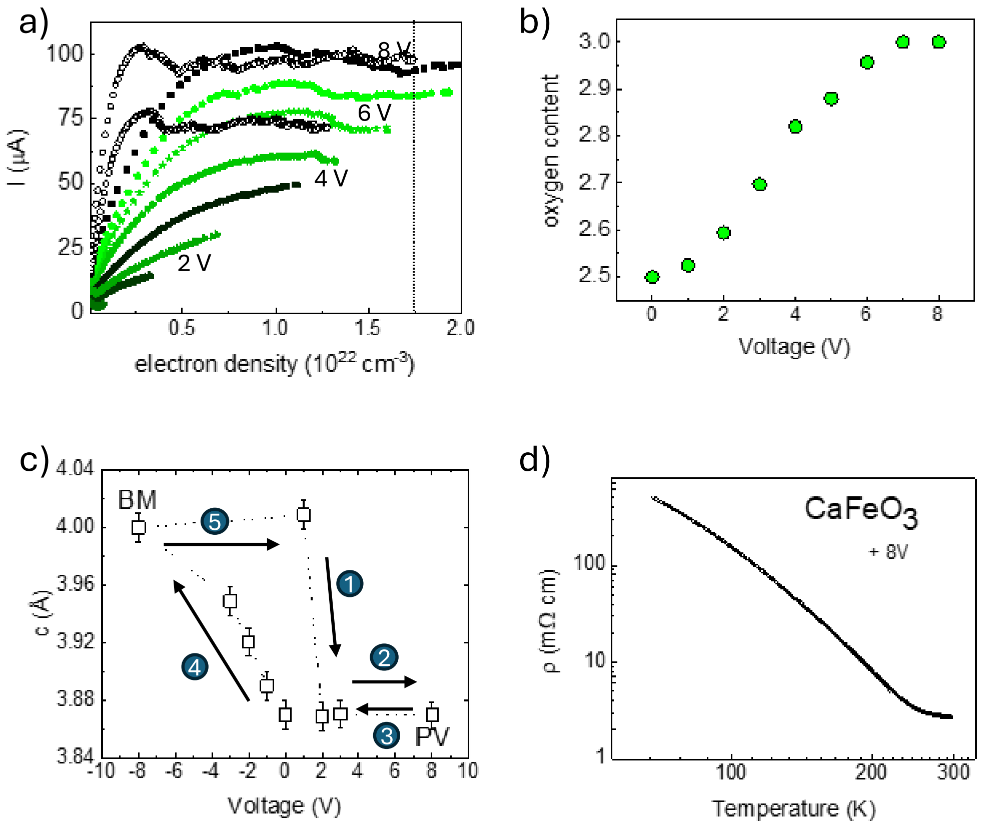

Figure S7. a) Current vs electron-density curves of the $CaFeO_x$ -CeO_2/YSZ film under different electric fields, according to $Q = (I \cdot t)/(e \cdot V)$, where I, t, e and V refer to the current intensity flowing through the sample, the application time of the voltage, the electron charge, and the volume of the film, respectively. The solid symbols correspond to films 40 nm thick and t=60 s; the open circles at 8 V and 6 V correspond to 140 nm thick and t=90 s. In b) we show the calculated oxygen content after one minute of redox reaction in the films 40 nm thick. Application of 8 V during 60 s (90 s) is enough to transform the 40 nm (140 nm) film of BM into a fully oxygenated PV. c) Evolution of the out-of-plane lattice parameter of the SFO film during the BM⇆PV transformation with the electric field. d) Temperature dependence of the electrical resistivity of the CFO film after oxidation to the PV with +8 V.

Table S1. Out-of-plane lattice parameters of the $CaFeO_x$ films oxidized/reduced under different conditions.

**$CaFeO_x$@STO**	**BM**	**PV (NaClO-ox)**	**BM-red**
c (Å)	3,685	3,777	3,696
**$CaFeO_x$@STO**	**BM**	**PV (O_3-ox)**	**BM-red**
c (Å)	3,685	3,761	3,692
**$CaFeO_x$@CeO_2@YSZ**	**BM**	**PV (+8 V)**	**BM (-8 V)**
c (Å)	3,901	3,778	3,901

Table S2. Out-of-plane lattice parameters of the $SrFeO_x$ films oxidized/reduced under different conditions.

**$SrFeO_x$@STO**	**BM**	**PV (NaClO-ox)**	**BM- red**
c (Å)	3,985	3,855	3,976
**$SrFeO_x$@STO**	**BM**	**PV (O_2-ox)**	**BM- red**
c (Å)	3,985	3,852	3,985
**$SrFeO_x$@CeO_2@YSZ**	**BM**	**PV (+8 V)**	**BM (-8 V)**
c (Å)	3,981	3,867	4,016

FDTR measurements.

The cross-plane thermal conductivity of the films was measured by Frequency Domain Thermoreflectance (FDTR).[5] FDTR is a non-contact optical pump–probe technique, in which one beam of light (the pump) acts as a heat source while a second beam (the probe) detects the resulting temperature change (ΔT) through a change in surface reflectivity (ΔR): $\Delta T = (dR/dT)^{-1}\Delta R = (\beta)^{-1}\Delta R$. The variable heat source produce temperature gradients and transients which makes it possible to measure κ and thermal boundary conductance with good precision. In our setup, a sinusoidal modulated pump laser (λ=405 nm, modulating frequency 2 kHz–50 MHz, Gaussian spot sizes $1/e^2$ radius ≈ 3.7 or 10.5 μm) is focused on the surface of the film, coated by a 60-nm-thick layer of Au, to produce an oscillatory modulation of the surface temperature. This results in a periodic variation of the Au thermoreflectance, which is probed by a laser beam (λ=532 nm). The probe beam is split before reaching the sample to work as a reference signal, improving the signal-to-noise ratio at low frequencies and compensating phase-shift offsets from beam paths and electronics. A similar setup is described in detail in reference[6]. The thermal properties of the sample are obtained by fitting the phase data to an analytical solution of the heat diffusion equation, in a multilayer model.[5] The thermal conductivity of the substrates was obtained from Ref.[7]; for Au we measured the electrical conductivity in co-deposited samples, and used the Wiedemann-Frantz law to obtain k_{Au}. The C_p of the substrate and Au transducer were measured or obtained from the literature. We performed sensitivity analysis for estimating the uncertainty of our FDTR measurements. We define the sensitivity of the phase signal to a fitting parameter α as (the phase in radians): $S(\alpha) = d\ \varphi / d\ln\alpha$.

To decouple the sensitivity for the parameters of interest ($\kappa\perp$, TBC1 and TBC2) we combined experiments with different spot sizes, and film thicknesses.

Table S3. Parameters used for the fittings of the FDTR data.

Layer	*Cp* (MJ/m³K)	κ (W/mK)	Thickness (nm)	TBC (MW/m²K)
Au	2.2	60-70	60	
CFO/SFO film	2.6	Fitting parameter	30-140 nm	67-100 (TBC 1)
STO substrate	2.67	9.8	5×10^3	500 (TBC 2)
YSZ substrate	2.8	2.2	5×10^3	

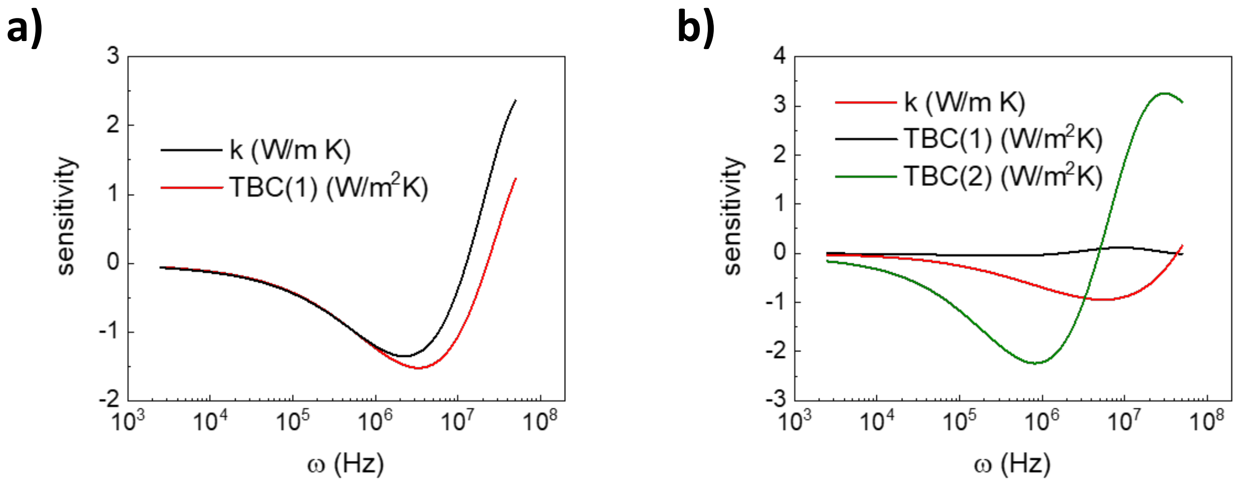

Figure S8. a) Sensitivity analysis to the thermal conductivity of a 40 nm thick film of CFO, and the interfacial thermal boundary conductance between Au/CFO. b) Similar calculations for a 140 nm thick film, which shows the decoupling of κ and the interfacial thermal conductance.

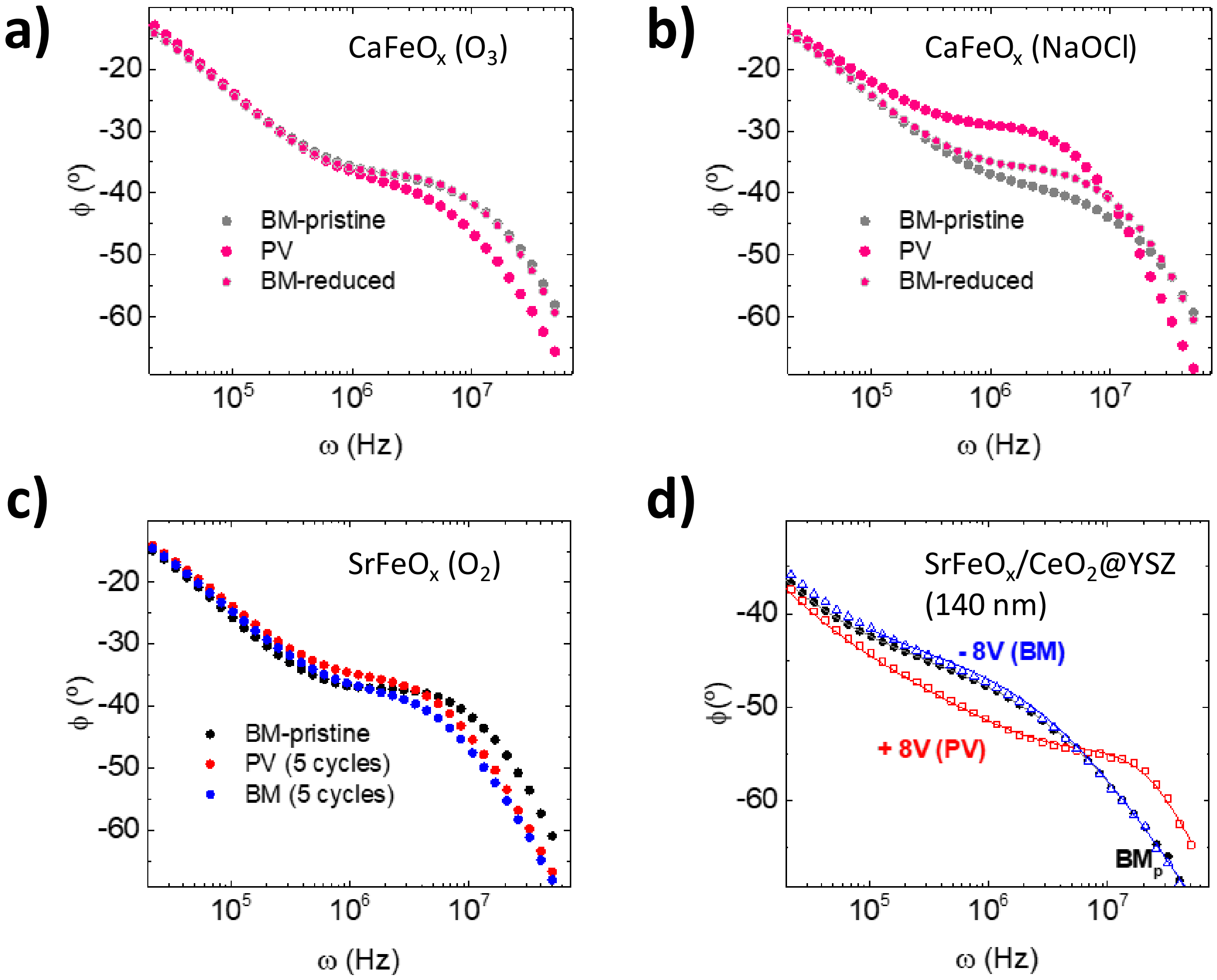

Figure S9. FDTR phase lag data for CFO oxidized in ozone (a) and NaOCl/H_2O (b). Panel b) shows that the irreversibility of BM→PV→BM process after wet oxidation is clearly reflected in the raw $\phi(\omega)$ data. In c) we show the curves after repeated oxidation/reduction of SFO in O_2, showing partial irreversibility also. The figure in panel d) shows the same data for a film transformed by electric-field-exchange of oxide ions with YSZ, showing the full reversibility of the process in this case. The solid lines are a fit to the thermal model to extract the thermal conductivity.

For avoiding any possible influence of deterioration of the Au transducer during the oxidation/reduction treatment of the samples, the Au transducer was deposited always after the chemical treatment. In the case of the samples transformed by an electric field on YSZ, we have observed only a minimum deterioration of the Au transducer. In this case, the results are comparable when a new Au layer is deposited after each transformation or if the transducer is maintained for several electric field cycles.

References:

(1) Tadano, T.; Gohda, Y.; Tsuneyuki, S. Anharmonic Force Constants Extracted from First-Principles Molecular Dynamics: Applications to Heat Transfer Simulations. *J. Phys. Condens. Matter* **2014**, *26* (22), 225402. https://doi.org/10.1088/0953-8984/26/22/225402.

(2) Tassel, C.; Pruneda, J. M.; Hayashi, N.; Watanabe, T.; Kitada, A.; Tsujimoto, Y.; Kageyama, H.; Yoshimura, K.; Takano, M.; Nishi, M.; Ohoyama, K.; Mizumaki, M.; Kawamura, N.; Fniguez, J.; Canadell, E. CaFeO2: A New Type of Layered Structure with Iron in a Distorted Square Planar Coordination. *J. Am. Chem. Soc.* **2009**, *131* (1), 221–229. https://doi.org/10.1021/JA8072269/ASSET/IMAGES/LARGE/JA-2008-072269_0009.JPEG.

(3) Dal Corso, A. Pseudopotentials Periodic Table: From H to Pu. *Comput. Mater. Sci.* **2014**, *95*, 337–350. https://doi.org/10.1016/j.commatsci.2014.07.043.

(4) Hibino, M.; Harimoto, R.; Ogasawara, Y.; Kido, R.; Sugahara, A.; Kudo, T.; Tochigi, E.; Shibata, N.; Ikuhara, Y.; Mizuno, N. A New Rechargeable Sodium Battery Utilizing Reversible Topotactic Oxygen Extraction/Insertion of CaFeOz ($2.5 \leq z \leq 3$) in an Organic Electrolyte. *J. Am. Chem. Soc.* **2014**, *136* (1), 488–494. https://doi.org/10.1021/JA411365Z/SUPPL_FILE/JA411365Z_SI_001.PDF.

(5) Schmidt, A. J.; Cheaito, R.; Chiesa, M. A Frequency-Domain Thermoreflectance Method for the Characterization of Thermal Properties. *Rev. Sci. Instrum.* **2009**, *80* (9), 094901. https://doi.org/10.1063/1.3212673.

(6) Yang, J.; Maragliano, C.; Schmidt, A. J. Thermal Property Microscopy with Frequency Domain Thermoreflectance. *Rev. Sci. Instrum.* **2013**, *84* (10). https://doi.org/10.1063/1.4824143.

(7) Langenberg, E.; Ferreiro-Vila, E.; Leborán, V.; Fumega, A. O.; Pardo, V.; Rivadulla, F. Analysis of the Temperature Dependence of the Thermal Conductivity of Insulating Single Crystal Oxides. *APL Mater.* **2016**, *4* (10). https://doi.org/10.1063/1.4966220.
